# Durability of single tablet regimen for patients with HIV infection in Southern Taiwan: data from a real-world setting

**DOI:** 10.1186/s12879-021-06919-6

**Published:** 2022-01-04

**Authors:** Hui-Min Chang, Chen-Hsi Chou, Hung-Chin Tsai

**Affiliations:** 1 Department of Pharmacy, Kaohsiung Veterans General Hospital, Kaohsiung, Taiwan; 2 Institute of Clinical Pharmacy and Pharmaceutical Sciences, College of Medicine, National Cheng Kung University, Tainan, Taiwan; 3 Department of Pharmacy and Master Program, Collage of Pharmacy and Health Care, Tajen University, Pingtung County, Taiwan; 4 Section of Infectious Diseases, Kaohsiung Veterans General Hospital, National Yang –Ming Chiao Tung University, Kaohsiung, Taipei, Taiwan; 5 Institute of Biomedical Sciences, National Sun Yat-Sen University, Kaohsiung, Taiwan; 6 Department of Parasitology, Kaohsiung Medical University, Kaohsiung, Taiwan; 7 Shu-Zen Junior College of Medicine and Management, Kaohsiung, Taiwan; 8 Section of Infectious Diseases, Department of Medicine, Kaohsiung Veterans General Hospital, #386 Ta-Chung 1st Road, 813 Kaohsiung, Taiwan

**Keywords:** HIV, Single tablet regimen, Antiretroviral therapy, Durability, Drug resistance

## Abstract

**Background:**

A single-tablet regimen (STR) has been associated with better drug adherence. However, the durability of different STRs was unknown in the real-world settings. Our aim was to investigate the durability of different initial STR regimens in antiretroviral-naive patients starting STR in southern Taiwan.

**Method:**

This was a retrospective study of antiretroviral-naive patients that initiated first-line antiretroviral regimens with STRs between May 2016 and December 2017. The primary endpoint was time to virological failure. Secondary endpoints were STR discontinuation due to toxicity/intolerance. Durability was defined as time from the initiation until discontinuation/modification. Kaplan- Meier curves were plotted assessing time to virological suppression, treatment failure and discontinuation for the three STRs and Cox proportional hazards model was used to analyze the factors associated with time to viral suppression, treatment failure or discontinuation.

**Results:**

Two hundred and twenty-three patients were included: The median follow-up duration (IQR) was 73.9 (48–101.6) weeks, 25 patients (11%) experienced virological failure; the 48 weeks probability of treatment failure was 22.9% (16/70) for Efavirenz/Emtricitabine/Tenofovir Disoproxil Fumarate (EFV/FTC/TDF), 24.1% (13/54) for Emtricitabine/Rilpivirine/Tenofovir Disoproxil Fumarate (FTC/RPV/TDF) and 24.2% (24/99) for Abacavir/Dolutegravir/Lamivudine (ABC/DTG/3TC) (p=0.16). Fifty-six patients (25%) discontinued their STRs owing to toxicity/intolerance. When compared to EFV/FTC/TDF, treatment with FTC/RPV/TDF (aHR 8.39, CI 1.98–35.58, p = 0.004) and ABC/DTG/3TC (aHR 8.40, CI 2.39–29.54, p=0.001) were more likely to have treatment failure. The predictors for treatment failure included age ≦ 30 years old (aHR 3.73, CI 1.25–11.17, p = 0.018), switch between different STR (aHR 2.3, CI 1.18–4.50, p  = 0.001) and free of active syphilis infection (aHR 0.24, CI 0.08–0.73, p = 0.012). The risk factor for treatment discontinuation included younger age ≦ 30 years old (aHR 3.82, CI 1.21–12.37, p = 0.023), treatment with EFV/FTC/TDF (aHR 8.65, CI 2.64–28.39, p < 0.001) and free of active syphilis infection (aHR 0.16, CI 0.04–0.62, p = 0.006).

**Conclusion:**

Younger age was associated with treatment failure and drug discontinuation. Active syphilis infection s/p treatment was associated with free from treatment failure and discontinuation. This probably driven by the more frequently sexual health education and counseling when patients had syphilis infection. Treatment with ABC/DTG/3TC was associated with higher risk of treatment failure. The STR durability was dependent on the drug toxicity/intolerance, age and syphilis infection.

## Background

Single tablet regimen (STR) has been associated with better drug compliance [1], improved quality of life [2] and less likely to development of resistance [3, 4] compared to multiple tablet regimens in antiretroviral therapy. Efavirenz/Emtricitabine/Tenofovir Disoproxil Fumarate (EFV/FTC/TDF, Atripla®) was the first STR in Taiwan and was introduced in 2010 then followed by Emtricitabine/Rilpivirine/Tenofovir Disoproxil Fumarate (FTC/RPV/TDF, Complera®) and Abacavir/Dolutegravir/Lamivudine (ABC/DTG/3TC, Triumeq®) in June 2016. The three STRs were recommended as the first line antiretroviral (ARV) drugs in Taiwanese reimbursement regulation for treatment-naïve patients with HIV infection since June 2016.

There are cohort studies dealing with ARV regimen durability in HIV infected treatment-naïve patients in the real-world settings. In a retrospective cohort study at eight UK centers, Lewis et al. found that the rilpivirine based STR (FTC/RPV/TDF) had a significantly lower discontinuation rate than another STR (EFV/FTC/TDF) and other third agents [5]. In another study from Maple Leaf Medical Clinic in Canada, researchers found that being on an integrase strand transfer inhibitor (INSTI) or nonnucleoside reverse transcriptase inhibitor (NNRTI)-based regimens versus protease inhibitors (PI) regimen were associated with longer time to virologic failure and better virologic durability. However, the information of durability of STR uses was lack [6].

The results with contemporary STRs in registrational studies in treatment-naïve HIV infected individuals showed that the efficacy was about 88–93%, rate of virological failure was 0.8–4.4% and rate of discontinuation due to severe adverse effects were around 0–1.9% [7]. Real- world ART durability may reflect more subjective treatment outcomes like quality of life or mild side effects that affect adherence in routine care and are likely of greater importance in clinic settings [8]. There is no pure STR durability data in the real- world.

The objectives of this study were to investigate the durability of different initial regimens in patients starting ART with single tablet regimen in southern Taiwan.

## Methods

### Study design and participants

This was a retrospective cohort study conducted at Kaohsiung Veterans General Hospital; a 1500-bed medical center in southern Taiwan. The study period was from May 2016 to Dec 2017. In Taiwan, all of the HIV infected patients were mandatory to enroll into the case management program sponsored by Taiwan Center for Disease Control (CDC) since 2008. The data of social, demographic, pharmacologic, laboratory, and concurrent infections were collected through electronic medical records. We included treatment-naïve patients with age more than 20 years old, initiating STR (one of the EFV/FTC/TDF, FTC/RPV/TDF, or ABC/DTG/3TC) between May, 2016 and December, 2017 in this retrospective cohort. This cohort study was conducted in parallel with the introduction of second and third STRs (FTC/RPV/TDF and ABC/DTG/3TC) into Taiwan in June 2016. Before June 2016, the only available STR was EFV/FTC/TDF which was used as the second line ARVs in those patients unable to tolerance to zidovudine (AZT)/3TC/EFV or HIV/HBV coinfection. After June 2016, any one of the three STRs (EFV/FTC/TDF, FTC/RPV/TDF, or ABC/DTG/3TC) was recommended as the first line therapy in those HIV-1 infected, treatment-naïve patients in Taiwan.

The laboratory tests for patients diagnosed with HIV infection included CD4 cell count, plasma viral load, serological markers for syphilis, hepatitis, cryptococcus, toxoplasmosis, cytomegalovirus, amoebiasis, liver and renal function. HIV-1 genotype resistance testing (GRT) for treatment-naive patients was not routinely performed in our country due to limited budget. Standard follow-up of the HIV-infected patients consisted of out-patient visits every 3 months. Testing for CD4 cell count, plasma viral load, haematology, and biochemistry were conducted every 6 months for viral suppressed patients. HIV Genotypic drug resistance testing for the protease and reverse transcriptase region was done by the commercial kit Viroseq version 2.8 (Celera; Quest Diagnostics, Secaucus, NJ, USA) [9]. For integrase sequencing, the in house protocol with nested reverse transcription polymerase chain reaction (RT-PCR) was used according to our previous published methods [10].

Durability was defined as the number of weeks from the STR regimen initiation until discontinuation or modification. Viral suppression was defined as viral load (VL) < 20 copies/ml. Virologic failure was defined if at least one of the following occurred: (1) failure to achieve viral suppression by 48 weeks or (2) after achieving viral suppression, having a detectable VL on two consecutive occasions at least 14 days apart or a single VL ≥ 200 copies/ml. Treatment failure was a composite endpoint defined as virological failure or discontinuation/switch of STR for any reason or death. Active syphilis was defined as a new positive rapid plasma regain (RPR) and the Treponema pallidum particle agglutination test (TPPA) [11]. In those patients had ever received syphilis treatment, a fourfold increase in the RPR titer was also indicated a new infection [12].

### Statistics analysis

Categorical data were analyzed using Chi-square test or Fisher’s exact tests as appropriate, and continuous variables, expressed as median and interquartile range, were compared using the Manne-Whitney U or Kruskal-Wallis test. Kaplan-Meier curves were plotted assessing time to virological suppression, treatment failure and discontinuation for the three STRs. The median duration for durability was reported in weeks and compared across stratified STRs using the log-rank test. In treatment failure analysis, patients were censored if they were lost to follow-up, discontinuation/switch of STR for any reason or death. Association of various demographic characteristics with time to viral suppression, treatment failure or regimen modification/ discontinuation were evaluated using Cox proportional hazards model to estimate crude and adjusted hazard ratios (HRs), and the associated 95% confidence intervals (CIs) were estimated by multivariable regression models. The following clinically important variables were included: age, sex, HIV transmission risk factor, CD4 count, viral load, concurrent infection with hepatitis A, B, C and syphilis and individual STR. Variables that were significant in univariate analysis were considered for the multivariable model. A stepwise selection was used to select the covariates to be included in the final model using a p-value of 0.2 to enter the model and a p-value of 0.10 to remain in the model. We also examined all variables for correlation and interaction and excluded the variables that were highly correlated. Validity of the proportional hazards assumption was evaluated using Schoenfeld residuals. A two-sided *p *< 0.05 was considered statistically significant. Statistical calculations were performed using the SPSS program version 12.0 (SPSS Inc., Chicago, IL).

## Results

### Patient characteristics

**Fig. 1:**
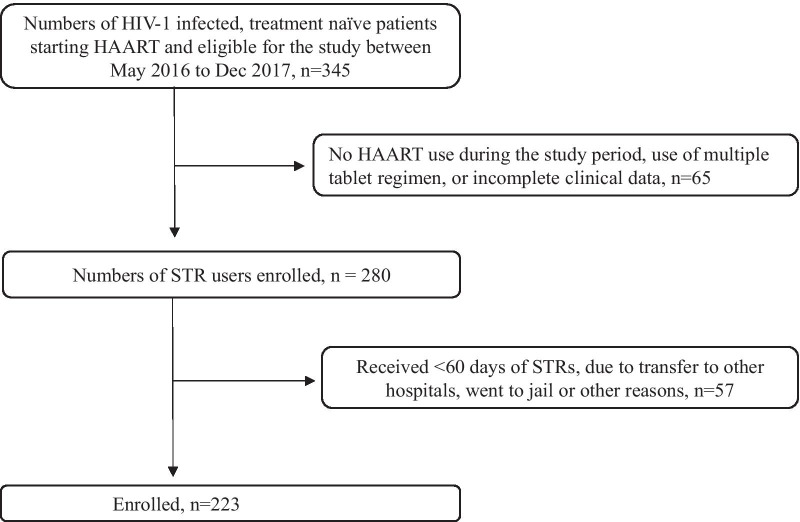
Study flow. HAART retention rate, adverse effects, and virological outcomes among HIV-1 infected treatment- naïve patients starting STRs

**Fig. 2:**
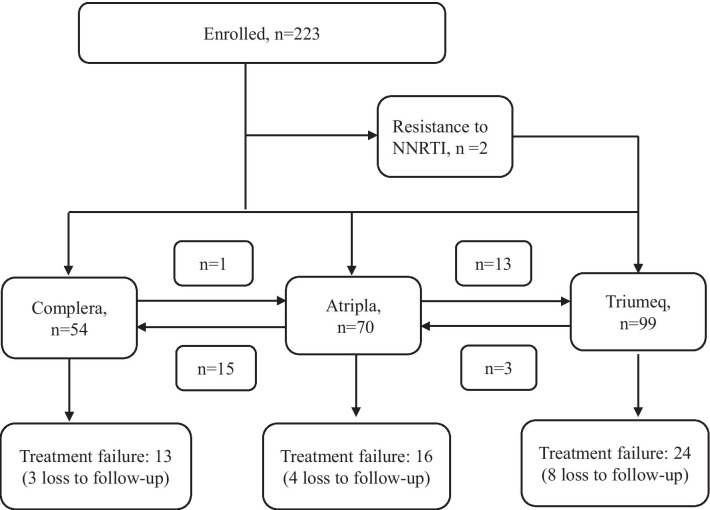
Treatment outcomes and drug switch among the 223 HIV-1 infected patients starting STRs

A total of 345 HIV-1 infected, treatment-naïve patients were eligible for the study between May 2016 to Dec 2017. Among them, 122 patients were not enrolled due to no ARV use during the study period, use of multiple tablet regimen, or incomplete clinical data, received with less than 60 days of STRs, transferred out or went to jail (Fig. 1). Baseline characteristics of the remaining 223 HIV-1 infected treatment-naïve patients starting STR between May 2016 to Dec 2017 were showed in Table 1. Briefly, their medium age (IQR) was 35 (27–41) years old with men consisting of 93.7%. The HIV transmission risk factor included MSM (67.7%) and intravenous drug abuser (26.5%). 26% (58/223) had concurrent infection with hepatitis C, 30.5% had hepatitis B, 48% hepatitis A and 41% syphilis. Only 27.3% (61/223) of the patients had baseline GRT. 31% (70/223) of the patients started EFV/FTC/TDF, 24% (54/223) FTC/RPV/TDF and 44% (99/223) ABC/DTG/3TC. The medium (IQR) duration of follow up (weeks) was 73.9 (48–101.6) weeks. Among the 223 patients starting STRs, 13 developed treatment failure to FTC/RPV/TDF (13/54, 24.1%), 16 had treatment failure to EFV/FTC/TDF (16/70, 22.9%), 24 to ABC/DTG/3TC (24/99, 24.1%) at week 48, without differences between groups (p = 0.16) (Fig. 2) The switch between different STR was also common. EFV/FTC/TDF was associated with significantly higher rates of treatment modification (50%, p < 0.0001) compared to FTC/RPV/TDF and ABC/DTG/3TC. Among the 70 patients starting EFV/FTC/TDF, 15 switched to FTC/RPV/TDF and 13 switched to ABC/DTG/3TC due to different adverse effects.

**Table.1:** Baseline characteristics of the 223 HIV infected treatment-naïve patients starting STRs

Characteristics	Total	Atripla		Complera		Triumeq		p-value
Frequency	223	70 (31.4)		54 (24.2)		99 (44.4)		< 0.001
Age, years^a^	35 (27–41)	33 (27–41.3)		39.5 (33.8–46)		33 (26–40)		< 0.0001
Sex^b^								
Male	209 (93.7)	66 (94.3)		49 (90.7)		95 (94.9)		< 0.0001
HIV risk factors^b^								
IVDU	59 (26.5)	14 (20)		32 (59.3)		16 (16.2)		0.009
MSM	151 (67.7)	50 (71.5)		17 (31.5)		71 (71.7)		< 0.0001
Heterosexual	13 (5.8)	5 (7.1)		4 (7.5)		10 (10.1)		0.196
Unknown	4	1 (1.4)		1 (1.9)		2 (2)		0.779
CD4 count at baseline^a^	288.75 (162–422)	253.8 (125–387)		350.7 (255–495)		265.7 (138.3–403.9)		< 0.0001
Last CD4 count^a^	495.2 (347–656)	485.8 (365.3–639.8)		497 (375.6–661.6)		518.4 (332.3–676.2)		< 0.0001
Viral load at baseline copies/ml^b^								
< 100,000	149 (66.8)	38 (54.3)		50 (92.6)		61 (61.6)		0.07
≧ 100,000	74 (33.2)	32 (45.7)		4 (7.4)		38 (38.4)		< 0.0001
Duration of follow-up (weeks)^a^	73.9 (48–101.6)	99.4 (55.8–125)		73.9 (37.2–96.9)		69.4 (45.3–86.3)		< 0.0001
Co-infections^b^								
Hepatitis A virus coinfection	107 (48)	36 (51.4)		25 (46.3)		46 (46.5)		0.045
Hepatitis B virus coinfection	68 (30.5)	21 (30)		25 (46.3)		22 (22.2)		0.82
Hepatitis C virus coinfection	58 (26)	16 (22.9)		28 (51.9)		14 (14.1)		0.05
Syphilis	92 (41.3)	32 (45.7)		19 (35.2)		41 (41.4)		0.02
GRT	61 (27.3)	28 (40)		5 (9.3)		28 (28.3)		< 0.0001
Virologic failure^b^	53 (23.8)	16 (22.9)		13 (24.1)		24 (24.2)		0.16
Switched another STR^b^	56 (25.1)	35 (50)		7 (13)		14 (14.1)		< 0.0001

GRT: genotype resistance testing; IQR: interquartile range; MSM: men who have sex with men; STR: single tablet regimen; IDU: intravenous drug users; Atripla: EFV/FTC/TDF; Complera: FTC/RPV/TDF; Triumeq: ABC/DTG/3TC

^a^Kruskal–Wallis test, median (IQR)

^b^Chi-square test, n (%)

### Evaluation of tolerability

A total of 56 patients (EFV/FTC/TDF 35, FTC/RPV/TDF 7, ABC/DTG/3TC 14) discontinued their STRs due to drug adverse effects (p < 0.0001). The most common cause for discontinuing EFV/FTC/TDF was neuropsychiatric adverse effects (30%, 21/70, p < 0.0001). The most frequent symptoms included insomnia and sleep disturbances as well as dizziness and dreaminess. For ABC/DTG/3TC, it was skin pruritis and rash (6%, 6/99). A similar but nonsignificant result was also seen in EFV/FTC/TDF (Table 2).

**Table 2:** Types of AEs leading to discontinuation among the 56 patients with HIV infections starting STRs

	Atripla (n = 70)	Complera (n = 54)	Triumeq (n = 99)	p-value^a^
Numbers (%) of AEs	35 (50)	7 (12.9)	14 (14.1)	< 0.0001
Neuropsychiatric (%)	21 (30)	1 (1.9)	0	< 0.0001
Skin (%)	6 (8.5)	2 (3.7)	6 (6)	0.319
Other (%)	8 (11.4)	4 (7.4)	8 (8)	0.449
Time to discontinue, median (IQR)	25 (14–57)	23 (5–14)	11 (5–31)	< 0.0.05

AEs: adverse effect; IQR: interquartile range; STR: single tablet regimen; Atripla: EFV/FTC/TDF; Complera: FTC/RPV/TDF; Triumeq: ABC/DTG/3TC

^a^Chi-square or Kruskal-Wallis test as appropriate

### Treatment response

**Fig. 3:**
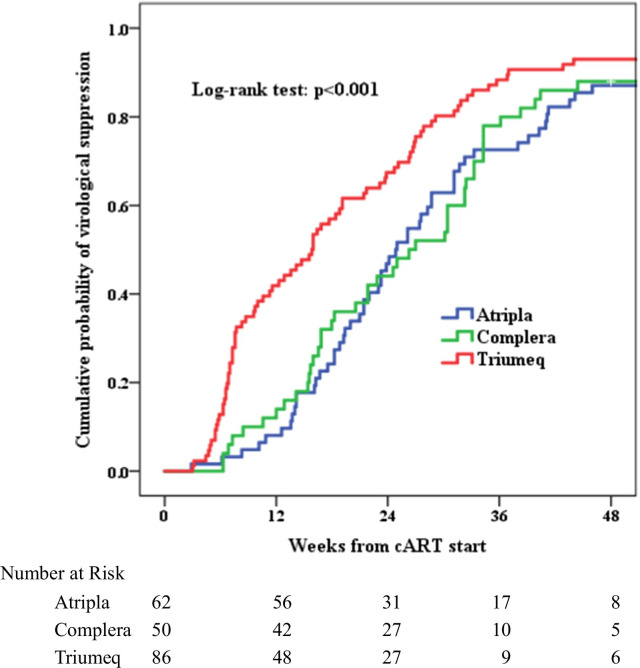
Time to viral suppression among 233 HIV-1 infected patients starting antiretroviral therapy with STRs (Atripla: EFV/FTC/TDF; Complera: FTC/RPV/TDF; Triumeq: ABC/DTG/3TC)

Time to viral suppression was different in 3 STRs. Compared to EFV/FTC/TDF and FTC/RPV/TDF, ABC/DTG/3TC was the ART with rapidly speed to reach viral load less than 50 copies/ml (15.86 weeks, CI 12.22–19.49), log rank test p < 0.001; Fig. 3). The predictors for viral suppression in the Cox proportional model included treatment with ABC/DTG/3TC (aHR 8.65, CI 2.64–28.39, p < 0.001), switch in different STRs (aHR 2.86, CI 1.47–5.52, p = 0.002) and free of active syphilis infection (aHR 0.15, CI 0.04–0.59, p = 0.006; Table 3).

**Table 3:** Predictors of virological suppression among 223 HIV infected treatment-naïve patients starting single tablet regimen

Variable	Comparator	Unadjusted hazard ratio (HR) (95% CI)	p-value	Adjusted hazard ratio (AHR) (95% CI)	p-value
Age ≦ 30 years	> 30years	2.71 (0.78 to 9.44)	0.12		
Baseline CD4+ count(cells/mm^3^) ≦100	> 100	1.33 (0.09 to 18.19)	0.83		
Treatment regimen^a^			0.003		0.002
	Atripla	1		1	
	Triumeq	9.85 (2.68 to 36.20)	0.001	8.65 (2.64 to 28.39)	0.000
HAV		1.49 (0.42 to 5.30)	0.53		
HBV		0.97 (0.19 to 5.05)	0.97		
HCV		0.71 (0.29 to 1.77)	0.47		
Switch between different STR		2.81 (1.26 to 6.25)	0.03	2.86(1.47 to 5.52)	0.002
Active syphilis infection		0.099 (0.02to 0.50)	0.005	0.15 (0.04 to 0.59)	0.006

CI: confidence interval; STR: single tablet regimen; Atripla: EFV/FTC/TDF; Complera: FTC/RPV/TDF; Triumeq: ABC/DTG/3TC; HAV: hepatitis A; HBV: hepatitis B; HCV: hepatitis C

^a^The HR and aHR of Complera in the subgroup analyses were less than 0.01. Complera was removed from the model due to small sample size

**Fig. 4:**
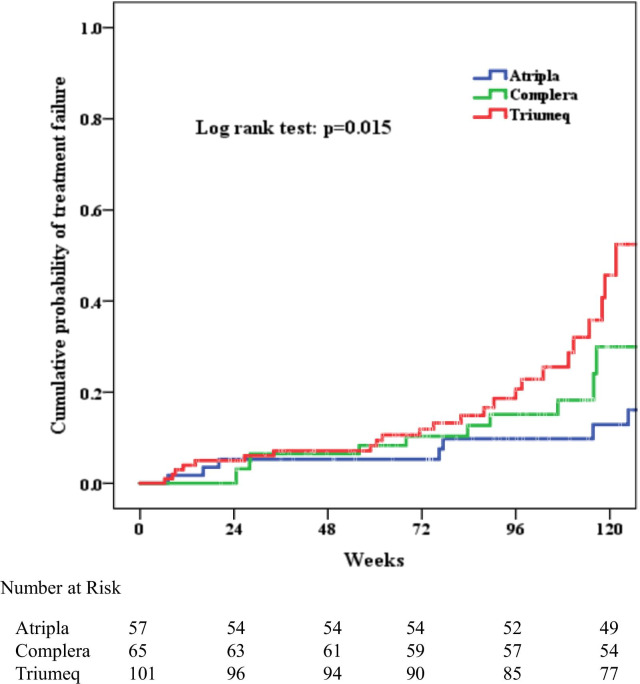
Cumulative probability of treatment failure among 223 HIV-1 infected patients starting antiretroviral therapy with STRs ( Atripla: EFV/FTC/TDF; Complera: FTC/RPV/TDF; Triumeq: ABC/DTG/3TC)

The risk of treatment failure was also different in 3 STRs (log rank test p = 0.015; Fig. 4). When compared to EFV/FTC/TDF, treatment with FTC/RPV/TDF (aHR 8.39, CI 1.98–35.58, p = 0.004) and ABC/DTG/3TC (aHR 8.40, CI 2.39–29.54, p = 0.001) were more likely to have treatment failure. When the risk of treatment failure was compared between two different STRs, treatment with FTC/RPV/TDF was not found to have higher risk of treatment failure when compared to EFV/FTC/TDF (log rank test p=0.116). However, treatment with ABC/DTG/3TC was associated with higher risk of treatment failure when compared to EFV/FTC/TDF (log rank test p = 0.048).

The other predictors for treatment failure included age ≦ 30 years old (aHR 3.73, CI 1.25–11.17, p = 0.018), switch between different STR (aHR 2.3, CI 1.18–4.50, p = 0.001) and active syphilis infection (aHR 0.24, CI 0.08–0.73, p = 0.012; Table 4).

**Table 4:** Predictors of treatment failure among 223 HIV infected treatment-naïve patients starting single tablet regimen

Variable	Comparator	Unadjusted hazard ratio (HR) (95% CI)	p-value	Adjusted hazard ratio (AHR) (95% CI)	p-value
Age ≦ 30 years	> 30years	4.985 (1.33 to 18.62)	0.017	3.73 (1.25 to 11.17)	0.018
Baseline CD4+ count (cells/mm^3^) ≦100	> 100	5.29 (1.55 to 18.02)	0.008		
Treatment regimen			0.003		0.002
	Atripla	1		1	
	Complera	7.83 (1.78 to 34.53)	0.007	8.39 (1.98 to 35.58)	0.004
	Triumeq	9.02 (2.40 to 33.95)	0.001	8.40 (2.39 to 29.54)	0.001
HAV		1.39 (0.51 to 3.80)	0.53		
HBV		0.90 (0.20 to 4.04)	0.89		
HCV		0.36 (0.06 to 2.17)	0.27		
Switch between different STR		2.37 (1.08 to 5.18)	0.03	2.3 (1.18 to 4.50)	0.001
Active syphilis infection		0.19 (0.06 to 0.64)	0.008	0.24 (0.08 0.73)	0.012

CI: confidence interval; STR: single tablet regimen; Atripla: EFV/FTC/TDF; Complera: FTC/RPV/TDF; Triumeq: ABC/DTG/3TC; HAV: hepatitis A; HBV: hepatitis B; HCV: hepatitis C

The risk factor for treatment discontinuation in the Cox proportional model included younger age ≦ 30 years old (aHR 3.82, CI 1.21–12.37, p = 0.023), treatment with EFV/FTC/TDF (aHR 8.65, CI 2.64–28.39, p < 0.001) and active syphilis infection (aHR0.16, CI 0.04–0.62, p = 0.006; Table 5).

**Table 5:** Predictors of treatment discontinuation among 223 HIV infected treatment-naïve patients starting single tablet regimen

Variable	Comparator	Unadjusted hazard ratio (HR) (95% CI)	p-value	Adjusted hazard ratio (AHR) (95% CI)	p-value
Age ≦ 30 years	> 30years	4.85 (1.08 to 21.83)	0.04	3.82 (1.21 to 12.37)	0.023
Baseline CD4+ count (cells/mm^3^) ≦100	> 100	1.14 (0.07 to 16.97)	0.92		
Treatment regimen			0.004		0.002
	Atripla	23.97(3.72 to 154.47)	0.001	16.61 (3.41 to 80.88)	0.001
	Complera	9.32 (0.63 to 138.72 )	0.105	8.59 (0.67 to 109.6)	0.098
	Triumeq	1		1	
HAV		2.71 (0.782 to 9.32)	0.12		
HBV		0.67 (0.14 to 3.25)	0.61		
HCV		1.25 (0.38 to 4.14)	0.71		
Active syphilis infection		0.17 (0.04to 0.81)	0.03	0.16 (0.04 to 0.62)	0.008

CI: confidence interval; Atripla: EFV/FTC/TDF; Complera: FTC/RPV/TDF; Triumeq: ABC/DTG/3TC; HAV: hepatitis A; HBV: hepatitis B; HCV: hepatitis C

## Discussion

In this study, we found that STRs virological failure and treatment discontinuation was quite common in the real-world settings. A high percentage (25%) of the patients discontinued their STRs due to toxicity/intolerance. Treatment with ABC/DTG/3TC and FTC/RPV/TDF were associated with treatment failure. However, when the risk of treatment failure was compared between two different STRs, treatment with FTC/RPV/TDF was not found to have higher risk of treatment failure when compared to EFV/FTC/TDF. Treatment discontinuation was common in the EFV/FTC/TDF group. Younger age ≦ 30 years old was associated with treatment failure and drug discontinuation. However, active syphilis received treatment was not associated with treatment failure and drug discontinuation.

The finding of a higher treatment failure rate in the ABC/DTG/3TC group was interesting and the result of a higher drug discontinuation rate in the EFV/FTC/TDF group was quite similar to that reported in the literature [5].

In a retrospective study from Maple Leaf Medical Clinic in Canada, researchers enrolled 780 patients between January 2006 and June 2016 to investigate the virologic durability of first-line INSTI, NNRTI, or PI -based antiretroviral regimens. No information on the percentage of STR uses were lack. They found that being on an INSTI or NNRTI-based regimens versus PI regimen, frequent VL testing and longer duration on of ART were associated with better durability [6].

In an Italian ICONA cohort, they prospectively followed up patients with low CD4 count (less than 200 cells/mm^3^) and high viral load (> 5 log10 copies/ml) who started first line ART with boosted protease inhibitors (bPIs), NNRTIs or INSTIs to analyze the durability of their different regimen. They concluded that starting ART with NNRTIs versus starting with bPIs; and starting ART with InSTIs versus starting with NNRTIs were less likely to be associated with treatment failure. Therefore, the durability of InSTIs-based regimen was longer than that of NNRTI- and bPI-based regimens [13]. In another study also from ICONA cohort, researchers evaluate the durability of three INSTIs and two NRTIs in ART naive patients. Among the 2016 patients enrolled, a total of 167 patients experienced treatment failure; the 1-year probability of treatment failure was 6.5% for raltegravir, 5.4% for DTG and 6.7% for elvitegravir/cobicistat. No detailed information on STR use was provided in both studies. Sixty-eight patients (3.4%) discontinued INSTIs owing to toxicity/intolerance. In the real-life setting, INSTI-based regimens showed high potency and durability. DTG are associated with a lower risk of treatment failure [14].

In a retrospective study in a multisite cohort (CFAR Network of Integrated Clinical Systems) in USA, they integrated data from eight Centers for AIDS Research (CFARs), focusing on HIV infected patients initiating ART between 2007 and 2014. In that study, 59% of patients starting their ART with STRs (EFV/FTC/TDF (n = 2173) 40%, Elvitegravir/Cobicistat/FTC/TDF (n = 571) 11%, FTC/RPV/TDF (n = 336) 6% and ABC/DTG/3TC (n = 106) 2%)). The initial regimen was modified in 43% (2285/5373) of patients. The median durability for all regimens was 48.6 months. Female sex, intravenous drug use, and CD4 cell count less than 200 cells/µl were significantly associated with regimen modification. NNRTI and INSTI-based ART were most durable in this study [15].

The possible explanation for the higher treatment failure rate in the ABC/DTG/3TC group in this study might be related to the high rates with drug discontinuation and loss of follow up. Clinicians may tend to prescribe high genetic barrier STR in those patients with suspicious poor drug adherence and severe diseases. Among the 24 patients suffered from virological failure, 8 loss of follow up, 11 patients reattained viral suppression at the end of our follow-up and 5 had persistent viremia. Only 2 patients had genotypic drug resistance testing, none had INSTI resistance-associated mutations and one of them had resistance to NRTI and NNRTI. In addition, the median follow-up times were short in our cohort, at around 1.5 years. This raises the possibility that, for some ART regimens such as ABC/DTG/3TC, the lost follow-up rate may be higher in the first year of therapy due to drug adverse effects but reduced to a lower rate (for those remaining on therapy) for subsequent years on treatment. The low STR discontinuation in the FTC/RPV/TDF group was similar to the study from UK. In a retrospective cohort study at eight UK centers conducted between 2012 and 2015 with only 2 STRs in their centers, Lewis et al. [5] found that the RPV based STR (FTC/RPV/TDF) had a significantly lower discontinuation rate than another STR (EFV/FTC/TDF) and other third agents.

Active syphilis received treatment was associated with less treatment failure and drug discontinuation. Our study result was different from that from the France [16] and similar to the 2 studies from Canada [17, 18]. In a large HIV cohort in France, they showed that early syphilis was associated with a 2-fold increase in the risk of viral load elevation in the months after diagnosis, even in patients receiving effective antiretrovial therapy [16]. In another study of systemic review and meta analysis, researchers did not find any direct evidence about the effects of sexually transmitted co-infection on transmission from individuals on ART. Their data suggested that the average effect of STI co-infection (including syphilis) on HIV viral load in individuals on ART were unlikely to decrease the effectiveness of treatment as prevention [17]. Recently, Grewal et al. determined the effect of acute syphilis on virologic failure among 2632 virally suppressed HIV-infected MSM taking antiretroviral therapy in Ontario, Canada. They demonstrated that acute syphilis was not associated with virological failure among virologically suppressed MSM on ART [18].

In this study, younger age (≤ 30 years) was associated with treatment failure and drug discontinuation. Several large cohort studies also report that older age was associated with better treatment outcomes [19, 20], and less virological non-suppression [21].

No data about the level of adherence was available in this study due to the retrospective cohort design. However, all of the patients were educated about their adherence and cared by the case management nurses, suggesting that other issues than adherence can explain their treatment failure and drug discontinuation [21, 22].

The reasons why the median time (IQR) to discontinue STRs were longer in the EFV/FTC/TDF group compared to FTC/RPV/TDF and ABC/DTG/3TC groups were unclear. This was probably due to the neuropsychiatric adverse effects for EFV/FTC/TDF were well known by the doctors, care management nurses and patients themselves, and patients were well educated about the adverse effects before the EFV/FTC/TDF was prescribing.

Our study had some limitations. First, there was always the risk of bias due to unmeasured confounding, such as drug adherence levels, not reporting adverse effects and missing data, when using retrospective cohort data. Secondly, reasons for regimen selection were not reported, patients with presumed poorer adherence, loss to follow-up in the initial phase of treatment and higher viral load were more likely to be prescribed ABC/DTG/3TC (INSTI-based) and could impact our analysis. Another caveat was that we censored patients who were lost to follow-up. Such censoring could have led to bias in increasing rate of treatment failure. Finally, our study results could not be applied to those health care systems whom did not have HIV case management program and free STR available.

In conclusion, younger age was associated with treatment failure and drug discontinuation. Active syphilis infection was not associated with an increase risk of treatment failure and discontinuation. Treatment with ABC/DTG/3TC was associated with more treatment failure. The STR durability was dependent on the drug toxicity/intolerance, age and syphilis infection regardless of genetic barrier of antiretroviral regimen.

## Conclusions

Younger age was associated with treatment failure and drug discontinuation. Active syphilis infection s/p treatment was associated with free from treatment failure and discontinuation. This probably driven by the more frequently sexual health education and counseling when patients had syphilis infection. The STR durability was dependent on the drug toxicity/intolerance, age and syphilis infection.

## Data Availability

The datasets used and/or analyzed during the current study are available from the corresponding author on reasonable request.

## Abbreviations

ART: Antiretroviral therapy
Atripla: Efavirenz/FTC/TDF
CDC: Center for diseases control
CFAR: Centers for AIDS Research
CI: Confidence interval
Complera: FTC/RPV/TDF
GRT: Genotype resistance testing
HAART: Highly active antiretroviral therapy
HAV: Hepatitis A
HBV: Hepatitis B
HCV: Hepatitis C
HR: Hazard ratio
IDU: Intravenous drug abuser
INSTI: Integrase strand transfer inhibitor
IQR: Interquartile range
VL: Viral load
MSM: Men who have sex with men
NNRTI: Nonnucleoside reverse transcriptase inhibitor
PI: Protease inhibitor
RCT: Randomized control trial
RPR: Rapid plasma regain
RT-PCR: Reverse transcription polymerase chain reaction
STR: Single tablet regimen
TPPA: Treponema pallidum particle agglutination test
Triumeq: ABC/DTG/3TC
